# Epistemic Beliefs and Learners' Self-Efficacy as Predictors of Language Learning Strategies: Toward Testing a Model

**DOI:** 10.3389/fpsyg.2022.867560

**Published:** 2022-04-12

**Authors:** Shaghayegh Shirzad, Hamed Barjesteh, Mahmood Dehqan, Mahboubeh Zare

**Affiliations:** ^1^Department of English Language and Literature, Islamic Azad University, Ayatollah Amoli Branch, Amol, Iran; ^2^Department of English Language and Literature, University of Mazandaran, Babolsar, Iran

**Keywords:** EFL learners, epistemic beliefs, language learning strategies, learners' self-efficacy, structural equation modeling

## Abstract

Understanding the beliefs held by the learners about learning a language, and the way they utilize their thoughts about knowledge and learning seem essential for planning a constructive language program. Following this line of research, this paper aims at testing a hypothetical model of the relationship between epistemic beliefs (EBs) and subscales of language-learning strategies (LLSs) through the mediating role of learners' self-efficacy (LSE). To this end, a sample of 300 Iranian high school students, taking regular courses, completed three survey questionnaires. At this stage, correlational analysis and structural equation modeling (SEM) were employed to probe the interconnections, analyze the model, and outline the conceptual framework. The results revealed that the LSE framework can adequately account for the learners' LLSs. In particular, the results indicated that efforts, persistence, and imitation (i.e., the subfactors of LSE) positively and significantly influenced LLSs. However, EBs with the mediating role of LSE were known to be a significant factor in demoting the LLSs. Notably, knowledge and learning agents were the negative predictors of LLSs. This paper suggests that LSE has higher explanatory power than EBs in predicting LLSs. The findings of this study suggest that teachers and material developers should pay serious attention to the learners' self-efficacy as they were known to influence LLSs.

## Introduction

Drawing on the second language (L2) professional literature indicates a paradigm shift from a cognitive and process-oriented approach to a beyond method-based pedagogy. Kumaravadivelu's (2006) paradigm in education potentially invites the learners with knowledge, beliefs, attitude, and autonomy necessary to foster their language learning. In line with Kumaravadivelu, voluminous studies (e.g., Hofer, 2016; Griffiths, 2018; Chamot, 2019; Lindner and Retelsdorf, 2019; Shirzad et al., 2020) have stipulated that learning conception, learners' beliefs, thinking about the essence of knowledge, and learning are connected to language learning. Recently, different studies (e.g., Morris et al., 2017; Liu et al., 2019; Takeuchi, 2019; Cheng, 2020; Mercer and Dörnyei, 2020; Razmi and Jabbari, 2021) have released evidence that learners' beliefs influence the academic achievement. They have provided strong evidence to support the predictive effect of learners' beliefs in learning achievement and course satisfaction. The findings also indicated that there is a positive interplay between different dimensions of EBs with different fields in sociology, psychology, and education. In education, various constructs were significantly reported to connect EBs such as epistemological theories (Hofer and Pintrich, 1997), language achievement (Winberg et al., 2019), self-concepts, learning conception (Liu et al., 2019), self-efficacy and assessment (Cheng, 2020), personal beliefs (Mardiha and Alibakhshi, 2020), LLSs and motivational self-system (Shirzad et al., 2020), and perfectionism (Razmi and Jabbari, 2021) to name but a few. Moreover, the EBs have been explored in various correlational studies (e.g., Chan and Elliott, 2004; Liu et al., 2019; Winberg et al., 2019; Mardiha and Alibakhshi, 2020; Shirzad et al., 2020; Kärchner et al., 2021; Zhu et al., 2021). The findings of such empirical studies indicated that EBs correlated with different variables such as stability, contingency of self-esteem, academic achievement, regulatory focus, learning engagement, conceptions of teaching and learning, and L2 motivational self-system. The results substantiated that learners' EBs have predictive power in education, in general, and learning conception. Besides, some other studies (e.g., Schommer, 1990; Hofer and Pintrich, 1997; Hofer, 2016) endorsed that learners with a high level of EBs seemed to act differently in various aspects of language learning and learning conception.

As a complex multidimensional trait, EBs (i.e., views about the quality of knowledge and learning), and LSE (i.e., the tendency for initiating tasks, investing adequate effort to conduct activities, endurance and perseverance in facing difficulties) are among the important affective factors in educational psychology (Hofer and Pintrich, 1997; Deuling and Burns, 2017; Shirzad et al., 2020; Razmi and Jabbari, 2021). An individual's EBs depict the conceptions of his/her delineations of scientific knowledge and what it denotes. Greene et al. (2016) used the term *epistemic cognition* to imply how students gain, apprehend, justify, and utilize knowledge. They postulated that learners involve in epistemic cognition when they arouse self-beliefs about the essence of knowledge and knowing (i.e., epistemic beliefs). Hofer and Pintrich (1997) postulated that EBs are indispensable for learning conception and understanding within several domains and contexts. Another construct of the current study is self-efficacy beliefs. Bandura (1997) conceptualized *self-efficacy beliefs* as an “individual's belief in his or her own ability to organize and implement action to produce the desired achievements and results” (p. 3). It is classified as a *general* or a *specific belief*. The former concerns a general perceived ability to confront stressful conditions, while the latter deals with a particular context or situation (Bandura, 1997). This study concentrates on specific self-efficacy beliefs related to the academic field described as an individual's perceived abilities to manage various instructional areas and learning conceptions. For the current study, EBs and LSE have been used as independent constructs to predict LLSs. Various theoretical studies (e.g., Oxford, 2017; Cohen, 2018; Griffiths, 2018; Chamot, 2019) pinpointed that learning strategies are an affiliative factor in promoting language achievement. Besides, the findings of some empirical studies (e.g., Oxford, 2017; Habók and Magyar, 2018; Takeuchi, 2019; Shirzad et al., 2020; Razmi and Jabbari, 2021; Tang, 2022) corroborated that LLSs can help the learners apply their knowledge in a real-world context, gain knowledge, and achieve higher academic results eventually. Accordingly, different taxonomies of LLSs were proposed by the authorities in educational psychology (e.g., Oxford's direct and indirect strategies; O'Malley and Chamot's socio-affective strategies; Cohen's L2 learning and use strategies). They substantiated the notion that utilizing LLSs can influence the quality of knowledge and learning (i.e., EBs) and different psychological constructs such as self-beliefs, self-concept, self-efficacy, to name but a few.

Despite the enriched literature (e.g., Hofer, 2016; Yang et al., 2019; Cheng, 2020; Kärchner et al., 2021; Lonka et al., 2021; Razmi and Jabbari, 2021; Zhu et al., 2021) on the predictive power of self-efficacy beliefs and their influence on the learning conception, the effects of EBs and LSE on the students' learning strategies are not yet clear. Accordingly, there has been no credible empirical study to support the conceptual interplay between EBs and LLSs with the mediating role of LSE. Therefore, it seems important to test a model to uncover how the learners' knowledge of EBs and their specific self-efficacy in the academic setting may predict the LLSs language learners employ in the learning process. Notably, it is significant to explore whether the dimensions of EBs promote or demote the learning strategies students utilize for language learning. Accordingly, it has been hypothesized that EBs and LSE promote students' learning strategy which in turn may foster their academic achievement. Moreover, it has been hypothesized that students' EBs and LSE positively predict high school students' LLSs. Despite sufficient evidence to support the positive effect of learners' beliefs, this paper claims that the target variables (i.e., EBs, LLSs, and LSE) have a complex and unpredictable relationship. Therefore, this study hypothesized a model based on the learners' beliefs and their sense of efficacy as the predictors of LLSs. Notably, this study was guided by the following objectives:

- To identify the relationship among the students' EBs, LLSs, and LSE.
- To determine whether EBs with the mediating role of LSE positively predict the high school students' LLSs.
- To explore if LSE positively predicts the high school students' LLSs.

## Literature Review

### Theoretical Framework

The professional authorities in Epistemology (e.g., Perry, 1970; Schommer, 1990; Magolda, 1992; Hofer and Pintrich, 1997; Hofer, 2001) classified two aspects for the EBs studies. They distinguished both *developmental* and *multidimensional* facets. The first aspect (i.e., developmental) is one-dimensional. Therefore, learners move through a cycle of developmental phases (i.e., from the objectivist view of knowledge to uncertainty of knowledge and then to extreme subjectivity). Notably, individuals move through the successive stages like fashion, and they progress in a patterned sequence of developmental stages. Hofer (2001) assumed that the thinking in this model opens with the objectivist perspective of knowledge to extreme subjectivity. Perry's model 1970 and Magolda's (1992) model of EBs are instances of the developmental model. Hofer (1994) postulated the notion of EBs as developing step by step in a linear fashion from *dualism, multiplism, relativism*, and finally *commitment*. Besides, Baxter Magolda (1992) conceptualized EBs in four stages: (a) a*bsolute*, (b) *transitional*, (c) *independent*, and (d) *contextual knowing*. In contrast to the developmental model, Schommer (1990) suggested a multidimensional system. Schommer claimed that EBs approximately exist in *independent beliefs*. This delineates that one dimension may be developed but another aspect may be quite immature. Notably, there are different beliefs that may (not) develop coincidently. Schommer's model included five dimensions (e.g., *stability, structure, source, speed of acquisition*, and *control of acquisition*). Hofer and Pintrich (1997) criticized Schommer's model in that the model cares about the nature of learning and not the nature of knowledge and knowing. Thus, they proposed four dimensions including certainty (stability), simplicity (structure), source of knowing (authority), and judgment for knowing (evaluation of knowledge). This study, therefore, delimited its scope on the learners' EBs in the educational domain. Accordingly, Schommer's model was adopted for the study because the scope of this paper was concentrated on the nature of learning and the academic setting.

#### The Theoretical Connections Among EBs, LSE, and LLSs

A growing body of theoretical and empirical studies (e.g., Morris et al., 2017; Takeuchi, 2019; Cheng, 2020; Razmi and Jabbari, 2021) corroborated that the learners' beliefs and their conception of learning influence language-learning behavior. Various theoretical studies (e.g., Perry, 1970; Schommer, 1990; Hofer and Pintrich, 1997; Bandura, 2006) pinpointed that individuals' EBs and LSE play pivotal roles in the learning process. As individuals with various beliefs may adopt different learning strategies, it seems that the constructs (i.e., EBs and LSE) may affect the learning process. Therefore, students with different levels of EBs and self-efficacy may act differently in language learning. Accordingly, they may adopt various learning strategies as far as their levels of EBs and self-efficacy are concerned. Despite consensus on the implications of the beliefs held by the learners, the way they may influence language learning raised doubts among the practitioners. Accordingly, various taxonomies for LLSs (e.g., O'Malley and Chamot; Cohen; Oxford) and scales for EBs and LSE (e.g., Clarebout et al., 2001; Chan and Elliott, 2004; Rezaei, 2010) have been proposed by the authorities to conceptualize the way the targeted variables influence language learning for different cultural situations. Recently, some empirical studies have gained attention on the learners' internal factors in the learning process. They underscored the connection between LLSs in the light of voluminous affective factors like learners' self-efficacy (Cheng, 2020), learners' beliefs (Winberg et al., 2019), L2 motivational self-system (Shirzad et al., 2020), self-control depletion (Lindner and Retelsdorf, 2019), regulatory focus, and learning engagement (Liu et al., 2019). Such empirical studies developed the perspective about learning strategies, thinking process in learning, and internal forces in education. Notably, exploring the interplay among EBs, LSE, and LLSs and the way EBs and LSE may influence language learning can foster significant pedagogical implications. Such findings formed the theoretical underpinning of this study.

### Epistemological Beliefs

The term *epistemology* is an area in psychology that deals with reasoning, the essence of knowledge, and the ideas about knowledge (Hofer and Pintrich, 1997). Hofer (2016) presumed that studies in EBs concentrate on the way learners come to know, and the way students employ their thoughts about knowledge and know how to conceptualize their environment. Winberg et al. (2019) conceptualized EBs as the beliefs about the essence of knowledge, learning, and knowing. Despite the lack of agreed-upon implementation of EBs, some authorities (e.g., Hofer, 2016; Winberg et al., 2019) used a multilayered stage. In line with the different layers, Shirzad et al. (2020) introduced some terminologies (e.g., *epistemic cognition, epistemic cognition, epistemological resources, epistemological reflection, personal epistemologies, reflective judgment*) to refer to EBs in the L2 professional literature.

In line with Perry's (1970) *dualism* model, Schommer (1990) suggested different beliefs about the origin of knowledge. She maintained that authorities manipulate various aspects of the beliefs. To Schommer, the structure of knowledge is an isolated rather than interrelated fact. Schommer distinguished different dimensions. The first dimension was established as *simple knowledge* (i.e., isolated facts) in the L2 professional literature. The second aspect (i.e., the certainty of knowledge) considers knowledge as an *absolute* (i.e., certain) construct. Schommer (1990) coined the term *omniscient authority* for the certainty of knowledge. The third aspect (i.e., speed of acquisition) pinpoints learning as a prompt vs. a gradual process. Finally, the *control of acquisition* refers to the ability to learn as natural vs. being acquired. To Schommer, it is an *innate ability* where learners believe learning cannot be enhanced with instruction. Concerning the multidimensional nature of EBs, Hofer (2016) elucidated that EBs deal with various constructs such as the *source, justification, certainty*, and the *development of knowledge*. Hofer assumed that the *source of knowledge* is at the level of less complex beliefs that originate beyond the self and occupies an exterior authority. At more complex beliefs, it is made by the knower in interaction with the peer. The term *justification of knowledge* deals with the way individuals account for knowledge. At the lower levels, they employ authority or observation rather than experiments, data, and inquiry rules. The *certainty of knowledge* is the belief about the validity of knowledge ranging from a belief in a correct answer to complicated problems. Finally, the *development of knowledge* concerns knowledge progress. It considers science as an evolving subject.

### Self-Efficacy Theory

Bandura's (1986) *self-efficacy theory* illustrates a picture of the learners' activity in which they are neither unlikely controlled by external factors nor automatically shaped by their genetic faculty. Bandura assumed that self-efficacy is an assumption in one's ability to conquer essential life events. In his theory, Bandura (1997) defined LSE as “people's judgments of their capabilities to organize and execute courses of action required to attain designated types of performance” (p. 174). Notably, various cognitive, affective, and biological forces are reciprocally influential SET. Bandura proposed five human abilities (i.e., *symbolizing capabilities, forethought, self-regulatory and self-reflective potential*) as the cornerstone of social cognition. Thus, LSE affects *what learners select to do*, their level of *endeavor, persistence* in case of problems, and *subsequent performance* (Sherer et al., 1982). Later, Bandura (2006) conceived that learners have a self-system that helps them to manipulate their emotions, feelings, and actions. In line with this claim, Morris et al. (2017) concluded that self-reflective capability provides learners with the capacity to think and to influence their future behavior. Likewise, Cheng (2020) conceptualized the notion of LSE as the belief that individuals can optimize their learning performance by their psychological attempts besides the scaffolding received by their peers and teachers in the educational settings. Specifically, LSE serves as a *self-regulatory* function that affects the learners' cognition and actions (Liu et al., 2019).

Pajares (2007) expressed that LSE is regarded as an anticipation process within self-regulation models. Pajares believed that LSE is a personal and social construct because learners act both collectively and individually. He maintained that LSE has a proactive effect on performance and self-evaluative operations along with performance. He outlined three distinctive self-efficacy features: First, self-efficacy focuses on *perceived competence* to execute a task rather than on psychological traits. Second, LSE beliefs are *task-, domain-*, and *context-specific*. Third, LSE depends on the *mastery criterion* of performance instead of the normative criteria. Finally, LSE beliefs are mainly evaluated before engaging in a *specific task* or *activity*. Spratt et al. (2002) released a motivational construct for the term LSE which predisposes learners to autonomous behavior. In this line, Lindner and Retelsdorf (2019) postulated that LSE is a dynamic and accomplished belief system that alters in diverse tasks and situations. Bandura (1997) highlighted the impact of LSE beliefs in individual performance. Bandura maintained that “people's level of motivation, affective states, and actions are based more on what they believe than on what is objectively true” (p. 2). Therefore, the way the learners act can be anticipated by the conceptions they hold about their abilities than by what they are capable of performing. Schunk and Zimmerman (2007) believed that such a construct encompasses different facets such as *level, generality, and strength*. More precisely, the former concerns the difficulty level of a task. The latter relates the transferability of the learners' efficacy judgments on various activities such as different academic subjects.

#### Dimensions and Sources of Academic Self-Efficacy in the Learning Context

Bandura (1986) postulated that the learners assess their efficacy by analyzing information from various aspects such as *mastery experience, vicarious experience, social persuasion, and physiological* and *affective states*. Bandura (1986, 1997) called this sort of attainment “*performance accomplishments”* and “*enactive attainments*”. Pajares (2007) concluded that mastery experiences comprised the attainment of goals (i.e., accomplishment/attainment) *via* direct and personal action (i.e., enactive). *Vicarious experience* is deeply rooted in the social model. Bandura (1986) believed that this source of self-efficacy is influential for the development of LSE for the novel task. Bandura called this model a *coping model* that openly struggles to overcome obstacles (Cheng, 2020). Later, Bandura (2006) highlighted the role of *evaluative feedback*. To him, it is a form of social persuasion that is often conciliated by perceived knowledge. Bandura maintained that the learners' self-beliefs may be more harmed by disappointing messages than influenced by positive conviction. He highlighted the roles of the physiological and affective states as the leading sources of LSE. Cheng (2020) stated that the notion of LSE concerns various self-beliefs, such as self-esteem, self-regulation, self-concept, and self-control. However, LSE is different from other kinds of self-beliefs (i.e., self-concept, self-esteem, and self-efficacy) that are mistakenly used interchangeably.

Bandura (1986) defined self-concept as a generalized self-assessment comprising different self-feedbacks such as feelings of self-worth and general competence beliefs. On the other hand, self-efficacy beliefs are context-specific judgments of individual capacity to manipulate courses of action to achieve a specific objective (Liu et al., 2019). LSE focuses on the tasks and activities one can perform than more global assessments of self-concept. Therefore, LSE promotes academic performance both directly and indirectly by its role on a learner's self-concept (Cheng, 2020). Pajares (2007) supported that self-esteem refers to the assessment of self-worth, which relies on the way culture values the characteristics one possesses and how well one's behavior corresponds to the standards of worthiness. Kärchner et al. (2021) called self-esteem a personal judgment of worthiness. Different practitioners conceptualized the construct as a subjective experience with general, situational, and task levels to capture its multifaceted aspects.

### Hypothesized Model

**Figure 1:**
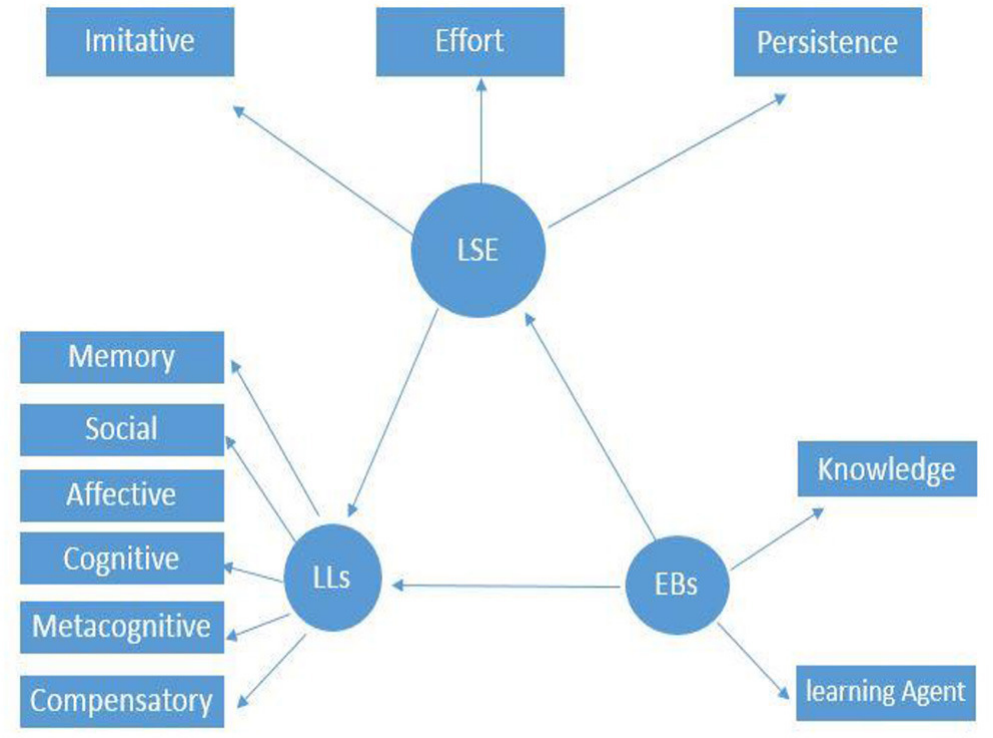
The hypothesized SEM model and the causal paths among the variables.

Drawing on previous theoretical frameworks, empirical studies, and justifying the connection among the targeted variables, this study proposed a structural model to determine the multivariate relations. The provided empirical evidence for the universal beneficial effects of EBs on academic achievement (Hofer, 2016; Peffer and Ramezani, 2019; Ongowo, 2021), and the supportive literature on the relationship between LSE and LLSs (Bandura, 1988; Pajares, 2007; Morris et al., 2017; Cheng, 2020) resulted in drawing a hypothesized path from the EBs to LSE and LSE to LLSs. For the current study, EBs and LSE are considered as independent variables, and the LLSs are regarded as dependent variables. To map the conceptual framework, a path diagram was generated based on the theoretical underpinnings to conceptualize a hypothetical model. At the theoretical phase, three constructs, measured by 11 observed variables, formulated the proposed model. Specifically, the hypothesized model predicts the path and the interconnections between EBs and LLSs with the mediating role of LSE. Following Fornell and Larcker's (1981) guidelines for generating a path diagram, latent (i.e., circles/ovals) and observed (rectangles) variables are illustrated in Figure 1.

Based on the hypothesized model and the interconnections proposed in the literature from the theoretical and empirical aspects among the variables, the following research questions were addressed.

RQ1: Is there any significant relationship among EBs, LLSs, and LSE?
RQ2: Do the learners' self-efficacy have a significant direct effect on their learning strategy?
RQ3: Do the students' epistemological beliefs have a significant direct effect on their learning strategy?
RQ4: Do the epistemological beliefs with the mediating role of the learners' self-efficacy have a significant indirect effect on LLSs among high school students?

## Methods

### Participants

To address the objectives of the current research, 300 Iranian high school students from a cluster of the entire population (*n* = 500) of Amol and Babol (i.e., two cities of Mazandaran) were recruited as the participants of this study. They were native Persian speakers who were both males (*n* = 123) and females (*n* = 177) with a similar language, social and cultural background. They were placed at the pre-intermediate level having 4 years of experience in learning English at different language institutes. To select a more representative sample, a cluster random sampling method was employed (Ary et al., 2018). The sampling multistage included two cities, five districts, and 12 state high schools. To control the bias effect, the respondents were randomly selected from two genders with different age ranges. Their ages ranged from 16 to 18 (M =17, SD = 1.7).

### Instrumentation

To collect the data, three scales were utilized regarding the target variables. Two questionnaires were translated into Persian and then back-translated by an expert translator to ensure the accuracy of the translation. To probe the translated versions, the original scales were examined by another expert holding a Ph.D. in Applied Linguistics. It was done to ensure the comprehensibility of the item, translation accuracy, and to check any ambiguities in comprehending the message. Next, the internal consistency was examined and reported. In addition, all the scales were piloted in a similar context. Specifically, to test the reliability of the scale within the EFL context of Iran, a pilot study was conducted among 120 pre-intermediated students learning English at three private English language institutes. The results enjoyed adequate reliability (α = 0.87).

#### Epistemological Beliefs Questionnaire

Razmi and Jabbari (2021) *EBQ* has been regularly administered as a well-known scale to appraise EBs. Originally, it comprised 63 items to be completed by the respondents. However, the appropriateness of this scale has been criticized for being long with confusing items. Accordingly, different adapted versions were validated in a setting different from the original one. For this study, an adapted version of the EBQ has been validated by Rezaei (2010). Rezaei examined the reliability and factor analysis of revised EBQ among 518 Iranian students studying different majors. To test the construct validity, exploratory factor analysis (EFA) and confirmatory factor analysis (CFA) were run. The EBQ scale enjoyed high reliability (α = 80.5) and validity indices. The revised EBQ comprised 16 items in either the negative or positive extreme. It aimed to measure the knowledge (*n* = 9 items) and learning agent (*n* = 7 items). The *first* dimension aimed to assess the respondents' assumption about the nature of knowledge (e.g., *If scientists try hard enough, they can find the truth to almost anything; wisdom is not knowing the answers, but knowing how to find the answers*). The second dimension concerned the learners' assumptions about acquisition/learning (e.g., *Learning something well takes a long time or much effort; How much you get from your learning depends mostly on your effort*). Students were asked to rate the statements on a five-point Likert scale from 1 (strongly disagree), anchoring the right end to 5 (strongly agree) anchoring the left end. In addition, the revised version was piloted for this study. The questionnaire was distributed among similar subjects (e.g., 100 junior high school students) in Amol and Babol, Iran. Some of them also joined the follow-up interviews to ensure the comprehension of all items. The scale enjoyed the total reliability coefficient (α = 0.77). The reliability coefficient for the subscale was as follows: simple/definitive knowledge (α = 0.78) and fast/fixed learning agent (α = 0.76).

#### General Self-Efficacy Questionnaire

The original GSEQ was developed by Sherer et al. (1982). It comprised 23 items with a construct of two factors (i.e., general and social self-efficacy). The general and social self-efficacy explained 26.5 and 8.5% of the variance. The alpha coefficient for the items is 0.86 and 0.70, suggesting that the items have a relatively high internal consistency. The GSEQ was arranged in a five-point Likert scale with 1 (not at all true) anchoring the left end and 5 (exactly true) anchoring the right end. The revised version comprised a construct of three factors: Initiative (9 items), Persistence (5 items), and effort (3 items). The GSEQ score ranged from minimum (17) to maximum (85). The score of the following items: 1, 13, 8, 9, 3, and 15, will increase from the right to the left, and the rest is *vice versa*. A higher score indicates a higher level of self-efficacy. To examine the internal consistency of the GSEQ in the EFL context of Iran, the questionnaire was piloted among high school students (*n* = 210) who were randomly selected from eight junior high schools. The estimated reliability was found to be (α = 0.796). Table 1 reports the reliability coefficient for each subscale.

**Table 1:** Cronbach's alpha coefficients for GSEQ.

**Sub-factor**	**Items**	**Alpha**
Initiative	9	0.84
Persistence	5	0.82
Effort	3	0.73
Total	17	0.796

As indicated in the table, all the subfactors (i.e., initiative, α = 0.84; persistence, α = 0.82; effort, α = 0.73) enjoyed a relatively high level of reliability.

#### Strategy Inventory for Language Learning

Oxford's (1990) SILL (version 7) was utilized to determine the frequency of LLSs. The SILL included 50 items in six subscales: (a) memory strategies utilized for storing and retrieving data (*n* = 9 items), (b) cognitive strategy employed for comprehension and production (*n* = 14 items), (c) compensation strategy aimed to address boundaries in linguistic knowledge/ performance (*n*= 6 items), (d) metacognitive strategy aimed to plan, organize, and monitor learning (*n* = 9 items), (e) affective strategy exploited to control motivation and emotion (*n* = 6 items), and (f) social strategies applied for interactive cooperation (*n* = 6 items). It employed a five-point Likert type ranging from 5 (always or almost always true of me) to 1 (never or almost never true of me). The score in a complete SILL ranged from 50 (minimum) to 250 (maximum). Different researchers (Griffiths, 2018; Habók and Magyar, 2018; Shirzad et al., 2020) reported the reliability coefficients for the SILL ranging from 0.85 to 0.98. Tahmasebi (1999) used CFA and EFA psychometric methods for validating and norming the translated version of the questionnaire for the Iranian students. The internal consistency of the scale was α = 0.91, suggesting that the translated version enjoys a relatively high internal consistency.

### Procedure

To collect the data, three questionnaires (i.e., *EBQ*, GSEQ, and SILL) were disseminated both through the survey link and through direct contact of the students. To undertake the study, the Google Docs application was utilized as a platform to create online questionnaires. Besides, some hard copies were distributed *via* the direct contacts of the researchers. Each questionnaire was distributed during regular class time. The students were asked to download each questionnaire and fill it out in the classroom, which took 15 min on average. They were asked to complete the questionnaires as meticulously as possible. To avoid fatigue, the instruments were administered at different intervals. The data were collected over 3 months in 12 weeks during the fall semester of 2019. A total of 240 students completed the questionnaires. All were completed to maximize the response rate (e.g., highlighting the benefits of the study, ensuring anonymity, providing reinforcement to respond). To minimize the bias effect, different high schools from three districts in two cities were randomly selected to distribute the questionnaires. After collecting the data, all the responses were screened for fact-checking to promote the veracity and correctness of reporting. Accordingly, a total of 130 questionnaires (29%) were not qualified for the analysis because they were incomplete or returned late. Specifically, 720 questionnaires (86%) met a valid response rate of 95%. Then, all the valid data were analyzed in the statistical package for the social science (SPSS) and the analysis of moment structures (AMOS) software using the SEM approach.

### Data Analysis

The data were analyzed using the SPSS version 22 and the AMOS version 20. The SPSS was employed to run descriptive statistics, and to check the normality of the data. The AMOS software was utilized to examine the probable structural relations between the independent variables (i.e., EBs, LSE) and the dependent variables (i.e., LLSs). SEM is a theory-driven and analytic procedure that provides the capability of path analysis to examine the interplay among various latent and observed variables with the capacity of factor analysis to ensure the construct validity of the factors and subfactors (Clark-Carter, 2010; Creswell, 2014). To run the SEM, both measurement and structural models are used. The former examines the interplay between a latent variable and its indicators. The latter checks the relation between the latent variables (Kline, 2011). Thus, CFA and expectation-maximization algorithm (EMA) were used to check the validity and missing items. Following Kline's guidelines, the goodness-of-fit indices (GFI) were utilized to examine the validity of the hypothesized model. The indices included χ^2^/df, GFI, comparative fit index (CFI), Tucker–Lewis index (TLI), and root mean square error of approximation (RMSEA). To check the parameters of distribution by promoting a likelihood function, maximum likelihood estimation (MLE) was utilized. Kline (2011) proposed that the values of these indices are acceptable if RMSEA <0.06, χ^2^/ df <3, CFI > 0.95, TLI > 0.95, and GFI > 0.95.

## Results

### Screening the Assumptions of Normality

To answer the research questions, some preliminary steps were taken to check the assumptions for normality of EBs, LLSs, and LSE. In so doing, skewness and kurtosis analyses of the target variables were run. Table 2 illustrates skewness, kurtosis, and normality analysis for the subscale of EBs, LSE, and LLS.

**Table 2:** Skewness, kurtosis, and normality test for different variables.

**Construct**	**Skewness**		**Kurtosis**		Kolmogorov Smirnov*	
	**SE**	**Statistic**	**SE**	**Statistics**	**Statistics**	**Sig**.
Knowledge	0.113	0.224	−0.162	0.364	1.695	0.006
Learning agent	0.302	0.224	−0.535	0.364	2.116	0.000
EBs	0.379	0.224	−0.077	0.364	1.529	0.019
Initiative	−0.040	0.224	−0.185	0.364	1.731	0.005
Effort	−0.115	0.224	−0.116	0.364	1.521	0.02
Persistence	0.180	0.224	−0.159	0.364	1.681	0.007
LSE	−0.151	0.224	−0.217	0.364	1.814	0.003
Memory	0.275		−0.222	0.364	1.134	0.153
Cognitive	−0.46	0.224	−0.175	0.364	1.090	0.186
Compensatory	−305	0.224	−0.525	0.364	1.136	0.151
Metacognitive	0.322	0.224	−0.639	0.364	1.023	0.246
Affective	−0.311	0.224	−0.506	0.364	2.056	0.000
Social	−164	0.224	0.345	0.364	1.702	0.006
LLS	−137	0.224	−0.173	0.364	1.361	0.049

* *This is a lower bound of the true significance*.

As indicated in Table 2, the distribution of data is normal, and the measure of skewness and kurtosis are at appropriate bounds for the different constructs. It implies that the values of skewness for the subfactors fall between – 3 and + 3, and kurtosis range from – 10 to + 10. Specifically, the measure of skewness (*range* = −0.040 to 0.379) and kurtosis (range = −0.077 to −0.639) are at appropriate bounds for the different subscales. In addition, the result of the KS test indicated that the data were not normally distributed (*p* > 0.05). To identify multivariate outliers, the Mahalanobis test was run. Mahalanobis distance (MD) determines the multivariate outliers. A maximum MD larger than the critical chi-square value (*p* < 0.001) for *df* = *k* (i.e., the predictor construct) shows the number of one or more multivariate outliers (Aryadoust and Raquel, 2020).

As indicated in Table 3 (*K* = 28.87; *df* = 19–1, *p* < 0.005), the minimum and maximum MD are 0.004 and 0.47, 0.341, respectively. The MD analysis indicates that 15 multivariate outliers do not match the general character of the dataset, and the total number of students (*n* = 258) falls within the normal range.

**Table 3:** Outlier detection with Mahalanobis distance.

	**Minimum**	**Maximum**	**Mean**	**SD**	***N***
MD	0.004	47.341	7.867	3.469	300
Leverage values	0.000	0.022	0.007	0.005	300

*MD, Mahalanobis' Distance*.

### Validation of Scales: CFA and Composite Reliability

**Figure 2:**
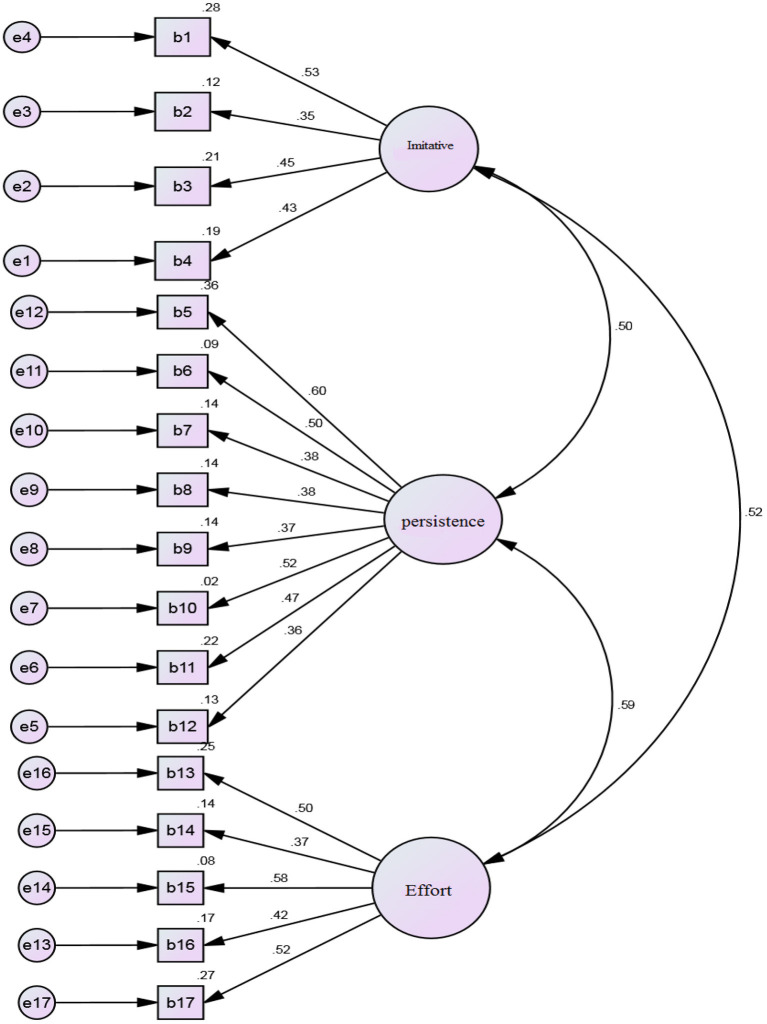
Standardized (β) coefficients for CFA analysis and error variance of LSE.

**Figure 3:**
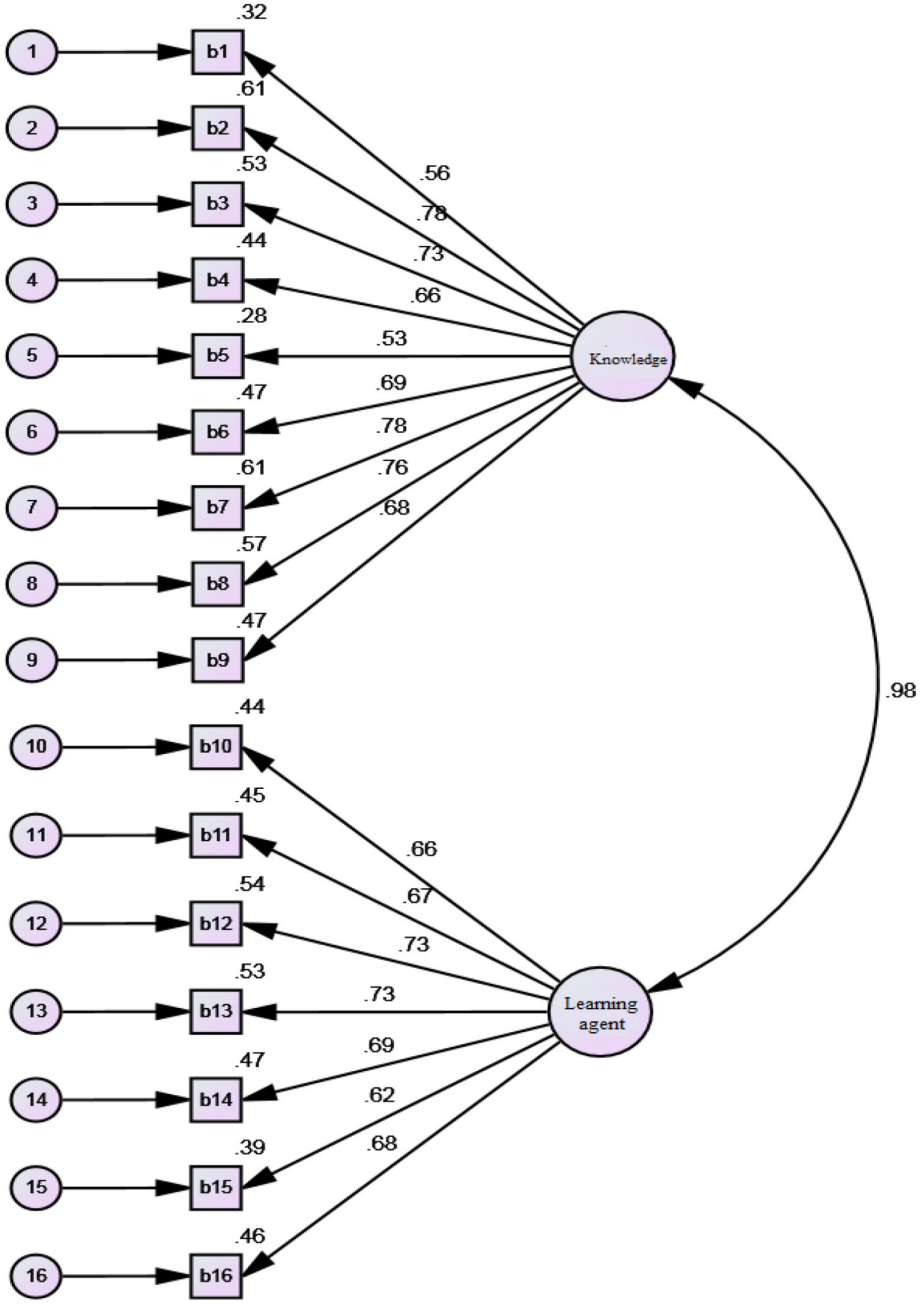
Standardized (β) coefficients for CFA analysis and error variance of EBs.

**Figure 4:**
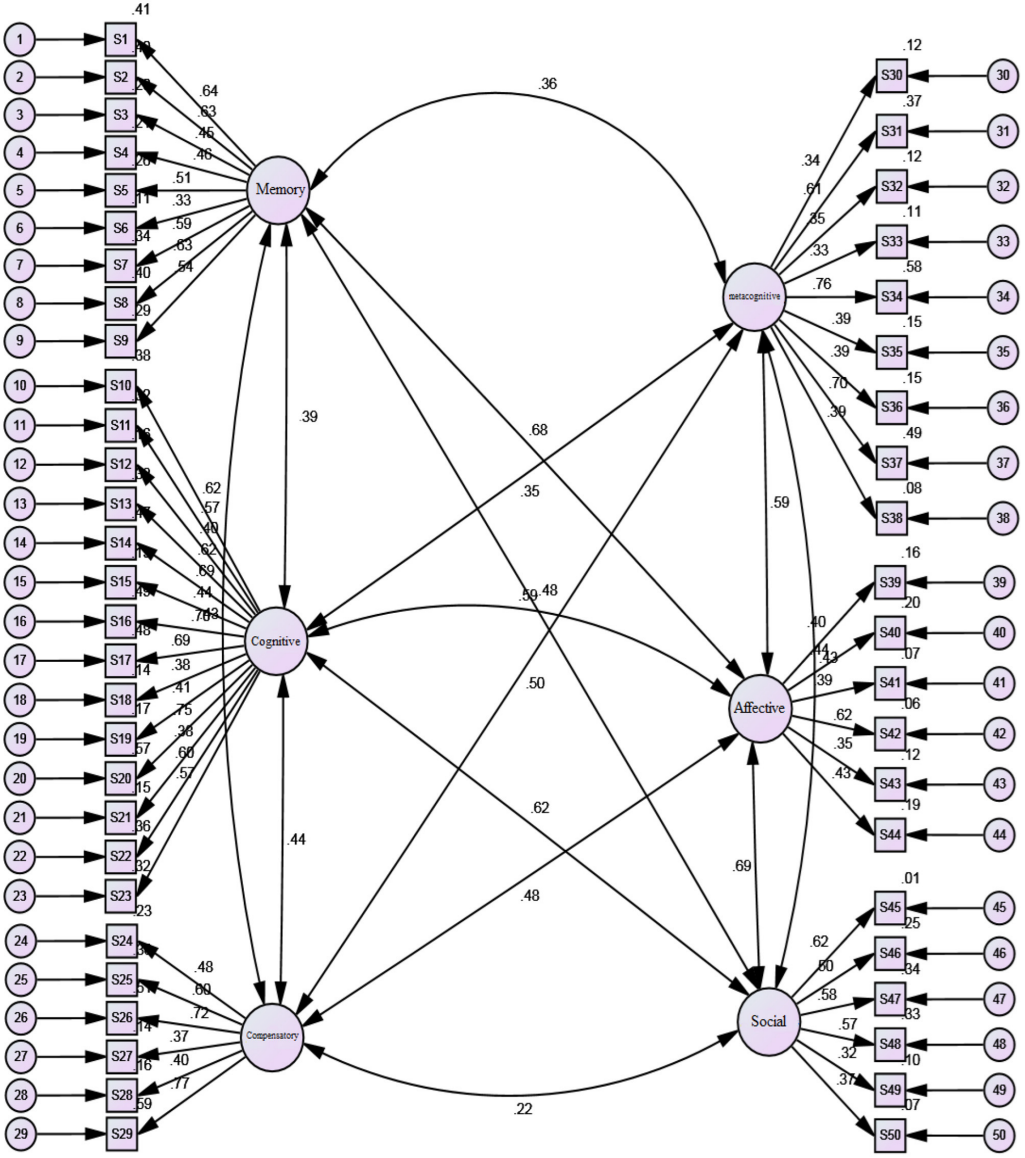
Standardized (β) coefficients for CFA analysis and error variance of LLSs.

To assure the construct validity of the instruments, CFA was run. The following models were designed and analyzed by the AMOS Graphics. Each figure schematically represents the standardized (β) coefficients for CFA analysis, different degrees of observed variables along standardized and unstandardized indices (see Figures 2–4).

The standardized (β) coefficients for CFA analysis indicate that all observed variables are above 0.30. Table 4 reveals that all observed variables for the different subscales fall within the acceptable model fit for LSE (i.e., *CFI* = 0.957; *df* = 104, *GFI* = 0.967, *TLI* = 0.972, *RMSEA* = 0.048), EBs (i.e., *CFI* = 0.954; *df* = 72; *GFI* = 0.93, *TLI* = 0.968, *RMSEA* = 0.031), and LLSs (i.e., *CFI* = 0.984; *df* = 24; *GFI* = *0.9*82, *TLI* = 0.985, *RMSEA* = 0.032). Thus, all the values illustrated for the model confirm that the factor loading of all the subscales for the variables are within the acceptable range and should be considered for the current study. Table 4 summarizes the confirmatory measurement model with the indicators.

**Table 4:** Conformity of measurement models with fitness indicators.

**Constructs**	**CFI**	**X2/df**	**GFI**	**TLI**	**RMSEA**	**Sig**.
LSE	0.957	104	0.967	0.972	0.048	0.000
EBs	0.954	72	0.93	0.968	0.031	0.000
LLSs	0.984	24	0.982	0.985	0.032	0.000

In addition, the convergent validity of the measurement model was examined by the average variance extracted (AVE) and composite reliability (CR). Following Fornell and Larcker's (1981) guidelines, the values of each indices are acceptable if AVE (*p* > 0.5) and CR (*p* > 0.7).

Table 5 indicates that the AVE and CR for all the components are above the criterion limit. Thus, it could be concluded that all questionnaires enjoy internal consistency as far as AVE and CR are concerned.

**Table 5:** Composite reliability for EBs, LSE, and LLSs.

**Construct**	**AVE**	**CR**	**Cronbach alpha**
Knowledge	0.723	0.839	0.745
Learning agent	0.521	0.770	0.786
EBs	0.539	0.766	0.824
Initiative	0.567	0.793	0.811
Effort	0.555	0.751	0.754
Persistence	0.590	0.801	0.796
LSE	0.53	0.916	0.854
Memory	0.555	0.895	0.752
Cognitive	0.553	0.832	0.798
Compensatory	0.527	0.810	0.731
Metacognitive	0.502	0.734	0.751
Affective	0.723	0.839	0.769
Social	0.572	0.759	0.824
LLSs	0.555	0.726	0.846

*AVE (p > 0.5); CR (p > 0.7)*.

To probe the interconnections among the main constructs (i.e., EBs, LLSs, and LSE), Pearson product-moment correlation was employed. Table 6 indicates the Pearson correlation matrix among EBs, LSE, and LLSs along with all the relevant subfactors.

**Table 6:** Pearson correlation matrix among EBs, LSE, and LLSs.

**F**	**M**	**SD**	**1**	**2**	**3**	**4**	**5**	**6**	**7**	**8**	**9**	**10**	**11**	**12**	**13**	**14**
1	45.97	5.87	1													
2	40.63	4.34	**0.82	1												
3	81.03	12.25	**0.62	**0.51	1											
4	11.82	2.45	**0.29	**0.33	**20.	1										
5	21.68	2.58	**-0.18	**-0.18	**-0.18	**0.52	1									
6	13.83	3.38	**-0.17	**-0.18	**-0.17	**0.49	**0.55	1								
7	41.18	5.04	**-0.20	**-0.19	**-0.21	**0.54	**0.67	**0.62	1							
8	24.38	3.67	**-0.19	**-0.19	**-0.17	**0.21	**0.16	**0.15	**0.19	1						
9	33.17	2.89	**-0.17	**-0.18	**-0.18	**0.19	**0.17	**19	**0.21	**0.51	1					
10	15.96	2.32	**-0.30	**-0.27	**-0.26	**0.31	**0.19	**0.20	**0.25	**0.43	**0.43	1				
11	19.74	1.23	**-0.26	**-0.26	**-0.21	**0.25	**0.17	**0.21	**0.24	**0.35	**0.50	**0.48	1			
12	18.06	1.45	**-0.23	**-0.20	**-0.22	**0.25	*0.10	**0.17	**0.22	**0.48	**0.52	**0.68	**0.51	1		
13	15.73	1.22	**-0.22	**-0.26	**-0.37	**0.53	**0.20	**0.22	**0.22	**0.66	**0.65	**0.68	**0.40	**0.63	1	
14	127.03	8.74	**-0.21	**-0.28	**-0.33	**0.60	**0.22	**0.22	**0.23	**0.74	**0.40	**0.46	**0.51	**0.46	**0.64	1

1. Knowledge; 2. Learning agent, 3. EBs; 4. Initiative; 5. effort; 6. persistence 7. LSE 8. Memory; 9. Cognitive; 10. Compensatory; 11. Metacognitive; 12. Affective; 13. Social; 14. LLSs. **P <0.01.

The results of descriptive statistics and Pearson correlation matrix among EBs, LSE, and LLSs indicate a significant correlation between two subfactors of EBs and three subfactors of LSE and LLSs. Notably, there is a significant negative interplay between EBs and LLSs. This implies that students with a higher level of EBs employ fewer learning strategies. Besides, there is a significant positive correlation between the total LSE and total LLSs. This shows that when language self-efficacy increases, students tend to use more learning strategies.

After conducting first-order CFA, the SEM approach was conducted to uncover the causal effects in the hypothesized model and to test the significance of the effects of the main latent variables. The second phase of the study was to examine if EBs and LSE significantly predict LLSs among high school students. Therefore, different fit indices were tested to evaluate the model fit. Table 7 indicates the GFI of the target variables.

**Table 7:** Goodness-of-fit indices of the EBs, LSE, LLSs.

**Fit index**	**Preference value**	**Obtained value** **before revision**	**Obtained value** **after revision**
*X^2^*/df	<3	3.042	2.847
*X^2^*	–	319.41	296.088
Df	–	105	104
RESMA	<0.1	0.051	0.041
AGFI	≤ 0.90	0.897	0.990
NFI	≤ 0.90	0.909	0.982
CFI	≤ 0.90	0.923	0.993

Table 7 indicates that RMSEA (0.051) lies within the preference value (*p* < 0.1). This value represents that the mean square error of the revised model falls within the acceptable fit threshold. Following the guidelines proposed by Aryadoust and Raquel (2020), all the fit indices (i.e., *AGFI* = 0.990; NFI = *0.9*82; *CFI* = 0.993; *df* = 2.847), enjoy the acceptable fit threshold. Accordingly, the revised measurement model was considered appropriate for further analysis. Table 8 indicates the regression analysis and coefficients for exogenous and endogenous variables. To determine the direct effect of EBs and LSE on LLSs, the MLE method was run. The MLE estimates the subfactors in distribution by maximizing a likelihood function (Richard, 2018). Table 8 indicates the result of the MLE for LLS.

**Table 8:** Direct maximum likelihood estimation for LLSs.

**Variable**	**Unstandardized coefficients**	**Standardized coefficients**	***R* ^2^**	***T***	**Sig**.
	**B**	**B**			
EBs	−0.482	**−0.380**	0.183	5.739	0.001
LSE	0.243	**0.167**	0.042	3.421	0.002

Table 8 indicates that the standardized coefficients of EBs (β = −0.380, *p* < 0.001) and LSE (β = 0.167, *p* < 0.001) are significantly predicted by LLSs. In addition, *R*^2^ for the EBs (*R*^2^ = 0.183) and LSE (*R*^2^ = 0.042) reveals that the conceptual model is statistically significant. To examine the indirect effects of EBs on LLSs with the mediating role of LSE, bootstrapping regression model was run. Table 9 reveals the bootstrap estimate of the indirect effect.

**Table 9:** Bootstrap estimate of indirect effect of EBs on LLSs with mediating LSE.

**Variable**	**B**	**Lower limit**	**Upper limit**	**Sig**.
EBs with mediating role of LSE on LLSs	0.441	0.260	0.497	0.000

**Figure 5:**
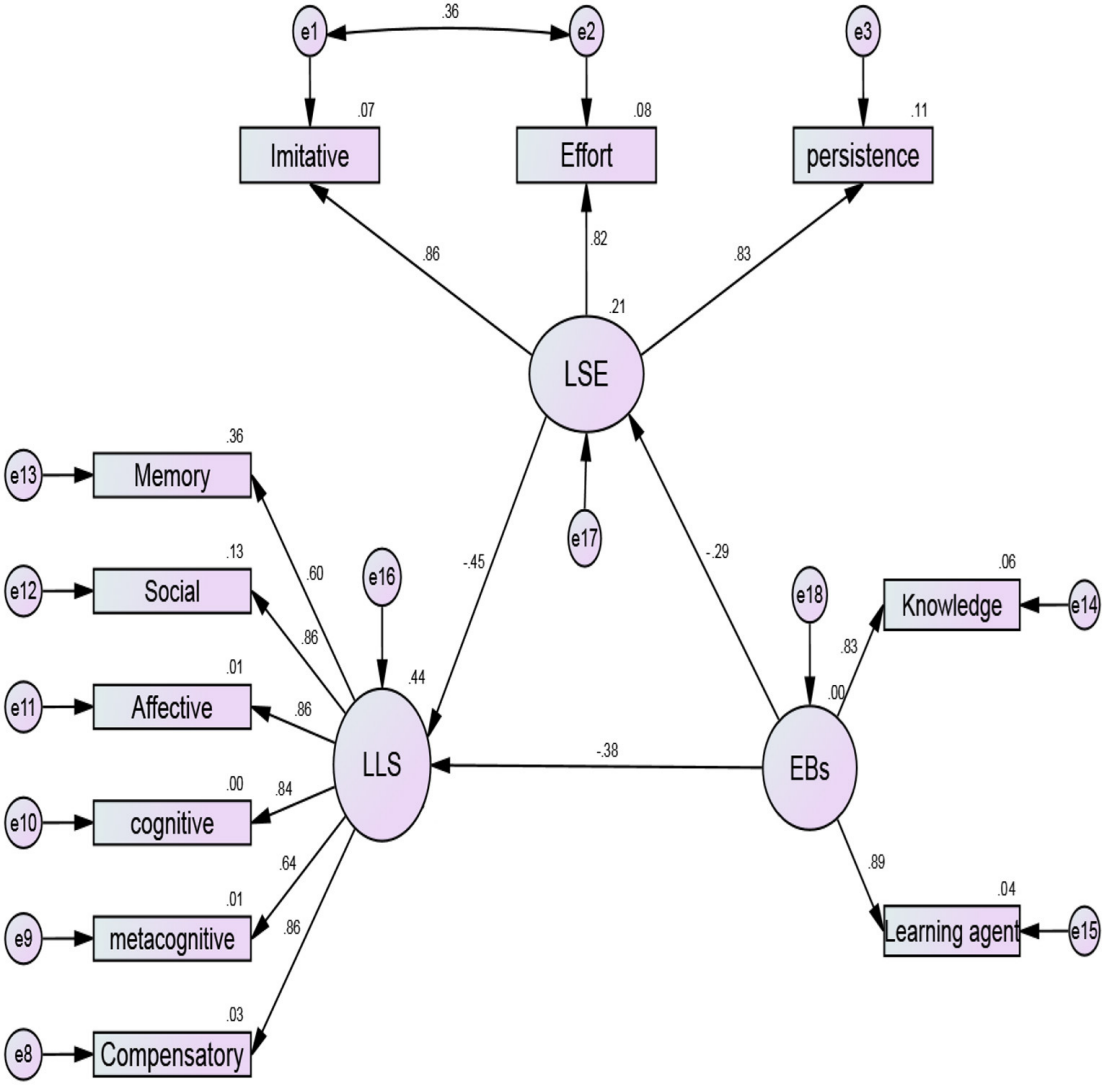
Standardized tested model and interrelationships among the EBs, LSE, and LLSs.

Table 9 indicates the standardized beta coefficients (β = 0.441, the lower limit = 0.260, upper limit = 0.422; *p* < 0.05). The result of the bootstrap for testing the indirect effect shows a significant level. In short, the path analysis for all direct and indirect effects predicts 34% of LLSs. The results show that EBs reduce LLS by 38% and LSE increases LLSs by 167%. Figure 5 indicates the interrelationship among the EBs, LSE, and LLSs.

## Discussion

The purpose of this study was to investigate the significance of the EFL learners' EBs and LSE in predicting their LLSs. To pursue the objectives, the interplay among EBs, LLSs, and junior high school LSE along with their components was examined using the SEM approach. The results of the SEM analyses indicated that the constructs (i.e., EBs and LSE) had a different contribution to LLSs. The standardized paths after correction for direct and indirect analyses revealed a direct and indirect effect on the EFL learners' LLSs concerning the exogenous factors (i.e., EBs and LSE). Notably, the results indicated that the subfactors of EBs and LSE significantly affect the subfactors of LLSs. The correlation coefficients among the two general variables of EBs and LSE were found to have significant positive and negative effects on LLSs.

The primary focus of this study was to probe whether EBs positively predict learners' LLSs. The path analysis of the hypothesized model revealed that EBs have a direct significant effect on LLSs. It has been hypothesized that EBs promote students' learning strategy which in turn may affect their academic achievement. The findings revealed that EBs with the mediating role of LSE on LLSs had a significant negative correlation with the subscales of LLSs. Notably, the findings suggested that EBs had an indirect effect on the types of strategy L2 learners may employ in the learning process. This finding implies that when students feel competent in their knowledge, they employ less learning strategy. The result echoes some theoretical studies (Bandura, 1997; Hofer and Pintrich, 1997; Hofer, 2016) that released evidence for the different aspects of EBs. The findings of such theoretical studies indicated a significantly strong correlation between the learners' LLSs and EBs. They postulated that some dimensions of EBs are negative predictors of academic achievement. They underscored that students are more unlikely to use different strategies when they show stronger beliefs in their knowledge and learning agent. Similarly, the findings corroborated the previous claims by L2 practitioners (e.g., Oxford, 2017; Griffiths, 2018; Chamot, 2019; Winberg et al., 2019; Tang, 2022). They corroborated that learning strategies are consistent with the beliefs held by learners. Different qualitative studies discussed in the literature review had also confirmed that the learners' beliefs (i.e., the beliefs about knowledge and learning) can influence the learning strategies their academic achievement (Habók and Magyar, 2018; Liu et al., 2019; Yang et al., 2019; Cheng, 2020; Mercer and Dörnyei, 2020). Such studies provided evidence that the level of the learners' beliefs affected their academic language achievement. Similarly, some authorities (Duell and Schommer-Aikins, 2001; Hofer, 2016; Griffiths, 2018) in the L2 professional literature highlighted that learning conception, thinking on the essence of knowledge, and learning strategies are interrelated with each other. Accordingly, they might have different positive and negative impacts on language learning. This study identified that the EBs influenced LLSs in a negative direction. The finding is consistent with Shirzad et al. (2020) corroborating that the level of the learners' EBs influence the type of strategies they utilize. Similarly, they concluded that the students who evaluate themselves as competent in their knowledge did not use strategy in terms of language learning. Therefore, it could be suggested that the higher the EBs, the fewer LLS would be employed in the learning process by language learners. In addition, the finding of the current study echoes the claim made by some practitioners (e.g., Sherer et al., 1982; Ekinci, 2017) that the learners' beliefs about their efficacy in language learning might affect their imitation, effort, and persistence. In other words, the high school students' beliefs in their competencies in employing appropriate strategies can predict their likelihood of effort, persistence, and imitation. To simply put, the EFL students who have stronger beliefs in their knowledge and learning agents are less likely to experience LLSs.

The findings of this study added to the claim in the L2 professional literature that when the learners imagine themselves as competent learners, they can use less learning strategy. The results echo Cohen (2018) and Hofer (2016) who highlighted the learners' beliefs and learning strategies. They underscored that the beliefs held by the learners and learning strategies play a pivotal role in the learning process. Specifically, the findings proved the theoretical underpinning that EBs can affect academic success. Accordingly, they corroborated Razmi and Jabbari (2021) model for the learners' beliefs in that EBs of the learners are in line with the learners' cognitive and affective factors. Notably, this finding supports Razmi and Jabbari (2021) theoretical claim that different aspects of the beliefs about the structure and source of knowledge affect the learners' academic achievement and psychological factors. Moreover, several qualitative studies, discussed in the literature review released evidence that the learners' beliefs promoted academic success (e.g., Morris et al., 2017; Lindner and Retelsdorf, 2019; Takeuchi, 2019; Mercer and Dörnyei, 2020). Similarly, some practitioners (e.g., Liu et al., 2019; Yang et al., 2019; Cheng, 2020) substantiated the predictive role of the learners' beliefs in the learning outcome and course satisfaction. They postulated that the beliefs held by the learners about knowledge and learning could affect their academic language achievement positively.

Another aspect of this study was to probe if LSE positively predicts their LLSs. Despite sufficient evidence to justify the positive effect of the learners' beliefs, this paper hypothesized that LSE may have a complex and unpredictable effect. Thus, it has been hypothesized that LSE promotes their learning strategy which in turn may foster their academic achievement. The path analysis of the hypothesized model revealed that LSE has a direct significant effect on LLSs. The analysis verified that all direct and indirect effects could account for 34% of the LLSs. The standardized tested model and correlation among the components of LSE and LLSs indicated that the two constructs (i.e., LSE and LLSs) correlated in a positive direction. Therefore, it could be postulated that when the students have a high level of academic self-efficacy, they use more learning strategies. It implies that the students who had a higher level of LSE seemed to employ greater LLSs. Moreover, some qualitative studies highlighted that self-efficacy positively correlated with the overall strategy use. Different studies (e.g., Bandura, 2006; Pajares, 2007; Lindner and Retelsdorf, 2019; Liu et al., 2019; Cheng, 2020) discussed in the L2 professional literature have also acknowledged a positive interplay between these two variables. The findings of the present study echoes different bodies of studies (i.e., Schunk and Zimmerman, 2007; Hofer, 2016; Osiochru, 2018; Winberg et al., 2019) and suggests that LSE was the robust predictor of LLSs. Moreover, the findings supported Bråten and Olaussen (2005, cited in Bandura, 2006) who specified that learners with higher levels of LSE seem to have higher knowledge beliefs. The result of the present study affirmed previous studies that learners who had a higher level of LSE also reported greater use of LLSs (e.g., Bandura, 2006; Pajares, 2007). The findings of this study added the claim in the literature that LSE might be increased teaching how to learn LLSs. Specifically, the findings reinforced the claim made by some authorities (e.g., Oxford, 2017; Cohen, 2018; Takeuchi, 2019) that learning strategies can promote different self-learning (e.g., Selfefficacy, self-regulated learning strategies, self-directed learning). Likewise, the findings support Cheng (2020) and Liu et al. (2019) who claimed that LSE serves a *self-regulatory* function. They postulated that LSE provides students with the ability to affect their cognitive processes and actions. Overall, the findings disclosed that LSE could affect LLSs in a positive direction and it could foster academic success in general. This finding might be a direction for future research.

## Conclusion

Considering the findings of the study, some pedagogical implications were made for those individuals who work as educational planners, teachers, and learners in the educational contexts. Furthermore, the results can be helpful for educational policymakers to review their educational policies and programs for teacher training according to the proposed model. Specifically, positive interconnections were observed between two subfactors of EBs and three subfactors of LSE concerning LLSs. Accordingly, a general conclusion for the current study is that incorporating a focus on learners' beliefs into L2 language learning can promote EFL learners' pedagogical efficacy in general. A straightforward implication of the study is that LSE should receive more attention from the language teachers and language policymakers. Notably, more attention should be given to fostering learners' beliefs, in general, and promoting LSE skills. Thus, L2 learners will not only get higher academic achievements, but may also be motivated in learning, develop autonomous learning, and self-regulate their academic activities. To put it simply, a distinct conclusion for this study is that the EFL students' EBs and LSE can affect their choice and application of LLSs. The findings illustrate that there is a negative correlation between EBs and LLS, and there is a positive causal relationship between LSE and LLSs. Notably, the model proposed in this study suggests that the higher the students have epistemic beliefs, the less likely they will adopt a wide range of LLSs. Besides, the more LSE they have, the more likely they will use LLSs. This reveals that the beliefs held by the learners and the level of LSE influence the type of strategies they adopt. The proposed model encourages material developers and school managers to consider learners' beliefs seriously to help students promote sophisticated beliefs about knowledge and learning agent. However, due to the limitations we encountered for collecting the data and selecting the subjects at the high school level, we may not generalize the findings to other contexts. Therefore, the current study could be replicated to investigate the level of EFL learners' EBs, reflective thinking, learning strategy use, and the contribution of EBs to their learning strategy use (i.e., cognitive, socio-affective, and metacognitive strategies). Besides, future studies may be directed if qualitative or mixed-method research designs with different validated scales are adopted to generalize the findings.

## Data Availability Statement

The raw data supporting the conclusions of this article will be made available by the authors, without undue reservation.

## Ethics Statement

Ethical review and approval was not required for the study on human participants in accordance with the local legislation and institutional requirements. Written informed consent for participation was not required for this study in accordance with the national legislation and the institutional requirements.

## Author Contributions

All authors listed have made a substantial, direct, and intellectual contribution to the work and approved it for publication.

## Conflict of Interest

The authors declare that the research was conducted in the absence of any commercial or financial relationships that could be construed as a potential conflict of interest.

## Publisher's Note

All claims expressed in this article are solely those of the authors and do not necessarily represent those of their affiliated organizations, or those of the publisher, the editors and the reviewers. Any product that may be evaluated in this article, or claim that may be made by its manufacturer, is not guaranteed or endorsed by the publisher.
